# Expression, Regulation and Putative Nutrient-Sensing Function of Taste GPCRs in the Heart

**DOI:** 10.1371/journal.pone.0064579

**Published:** 2013-05-15

**Authors:** Simon R. Foster, Enzo R. Porrello, Brooke Purdue, Hsiu-Wen Chan, Anja Voigt, Sabine Frenzel, Ross D. Hannan, Karen M. Moritz, David G. Simmons, Peter Molenaar, Eugeni Roura, Ulrich Boehm, Wolfgang Meyerhof, Walter G. Thomas

**Affiliations:** 1 University of Queensland, School of Biomedical Sciences, Brisbane, Australia; 2 German Institute of Human Nutrition (DIfE) Potsdam-Rehbruecke, Department of Molecular Genetics, Nuthetal, Germany; 3 Oncogenic Signaling and Growth Control Program, Research Division, Peter MacCallum Cancer Centre, Sir Peter MacCallum Department of Oncology, University of Melbourne, Melbourne, Australia; 4 Queensland University of Technology, Faculty of Health, School of Biomedical Sciences, University of Queensland, School of Medicine, Brisbane, Australia; 5 University of Queensland, Centre for Nutrition & Food Sciences, Queensland Alliance for Agriculture and Food Innovation, Brisbane, Australia; 6 Center for Molecular Neurobiology, Institute for Neural Signal Transduction, Hamburg, Germany; University of Tokyo, Japan

## Abstract

G protein-coupled receptors (GPCRs) are critical for cardiovascular physiology. Cardiac cells express >100 nonchemosensory GPCRs, indicating that important physiological and potential therapeutic targets remain to be discovered. Moreover, there is a growing appreciation that members of the large, distinct taste and odorant GPCR families have specific functions in tissues beyond the oronasal cavity, including in the brain, gastrointestinal tract and respiratory system. To date, these chemosensory GPCRs have not been systematically studied in the heart. We performed RT-qPCR taste receptor screens in rodent and human heart tissues that revealed discrete subsets of type 2 taste receptors (*TAS2*/*Tas2*) as well as *Tas1r1* and *Tas1r3* (comprising the umami receptor) are expressed. These taste GPCRs are present in cultured cardiac myocytes and fibroblasts, and by *in situ* hybridization can be visualized across the myocardium in isolated cardiac cells. *Tas1r1* gene-targeted mice (Tas1r1*^Cre^*/Rosa26*^tdRFP^*) strikingly recapitulated these data. *In vivo* taste receptor expression levels were developmentally regulated in the postnatal period. Intriguingly, several *Tas2r*s were upregulated in cultured rat myocytes and in mouse heart *in vivo* following starvation. The discovery of taste GPCRs in the heart opens an exciting new field of cardiac research. We predict that these taste receptors may function as nutrient sensors in the heart.

## Introduction

G protein-coupled receptors (GPCRs) are seven transmembrane-spanning proteins that mediate cellular and physiological responses by converting extracellular stimuli into intracellular signals. GPCRs represent the largest receptor superfamily in the genome, recognizing and binding an array of sensory input and ligands, including photons, ions, odors/tastes, bioamines, lipids, peptides and proteins [1]. Many of the ligands for these receptors, including norepinephrine/epinephrine, endothelin and angiotensin have profound homeostatic and regulatory effects on the cardiovascular system. Not surprisingly, mutations and modifications of GPCRs, G proteins and their regulatory partners are linked to dysfunction and disease, with an estimated 40% of all drugs on the market eliciting their activity through GPCRs [2]. Although cardiovascular therapeutics are well established clinically, they target a very small fraction of cardiac-expressed GPCRs. Moreover, conservative estimates are that the heart expresses upwards of 100 different nonchemosensory GPCRs, yet over 30% of these have no known endogenous ligand, indicating that much biology and many potential targets remain to be discovered [3], [4].

A case-in-point is the chemosensory (odorant and taste) receptors, which account for over half of the GPCR repertoire. Previously considered exclusive mediators of olfaction and taste, these large GPCR families have been generally neglected as drug targets, with the exception of the fragrance and food industries [1]. However, it is becoming evident that chemosensory receptors are expressed in diverse tissues, where they perform additional functions and could represent important therapeutic targets. For instance, in the mouth, the taste receptor type 1 (*TAS1* in humans; *Tas1* in rodents) family sense the nutrient content of food and mediate sweet (*TAS1R2-TAS1R3*) and umami (*TAS1R1-TAS1R3*) taste. In other organ systems, such as in the brain [5] and gastrointestinal tract [6], [7], [8], *Tas1* receptors have also been implicated in nutrient sensing and regulation of hormone release. In addition, intriguing recent work suggests that *Tas1* GPCRs act as direct sensors to communicate amino acid availability to the mammalian target of rapamycin complex 1 (mTORC1) and regulate autophagy [9].

The taste receptor type 2 (*TAS2*/*Tas2*) GPCRs encode a family of ∼30 highly divergent receptors that mediate bitter taste [1]. *Tas2r*s expressed in taste papillae in the tongue detect and respond to a large number of structurally diverse aversive and toxic compounds, resulting in canonical avoidance and rejection responses [10]. Beyond the mouth, the *Tas2rs* are enigmatic, having been implicated in several distinct functions in the airways and throughout the gastrointestinal tract [11]. Specifically, *Tas2r*s have been identified in ‘solitary’ chemosensory cells throughout the respiratory epithelium, as well as in the airway smooth muscle, where they might respond to bitter/toxic compounds to mediate protective airway reflexes and bronchodilation, respectively [12], [13], [14]. *Tas2rs* have also been identified in specialized subpopulations of gastrointestinal cells, where they modulate hormone release and impact on gastric emptying [15], [16].

The expression of taste GPCRs is yet to be systematically studied in the heart. Here, we report the expression of *TAS1/Tas1* and *TAS2*/*Tas2* GPCRs in human and rodent heart, their cellular localization, developmental regulation and upregulation in conditions of starvation. We speculate that one function of cardiac cells expressing taste GPCRs might be as sentinels for cardiac nutrient sensing.

## Materials and Methods

### Ethics Statement

Human hearts were obtained immediately following explantation from five patients with terminal heart failure undergoing heart transplantation at The Prince Charles Hospital, Brisbane. Written informed consent was obtained from each patient (ethics approval number EC28114, Human Research Ethics Committee, Metro North Hospital and Health Service, The Prince Charles Hospital).

All animal procedures were carried out with specific approval from The University of Queensland Animal Welfare Unit (ethics approval numbers SBMS/095/11/NHMRC/NHF and ANAT DVB/658/07NHMRC) and follow the Australian Code of Practice for the Care and Use of Animals for Scientific Purposes.

### Experimental Animals and Tissue Collection

Animals were maintained on a 12 hour light/dark cycle with *ad libitum* access to standard chow and water. Sprague-Dawley Rats were killed by intraperitoneal injection of an overdose of sodium pentobarbital. Tas1r1*^Cre^/*Rosa26*^tdRFP^* mice report the history of activity of the *Tas1r1* promoter. Briefly, Tas1r1*^Cre^* mouse line was generated by replacing the entire Tas1r1 coding region by an expression cassette containing the coding sequence of barley lectin, an internal ribosomal entry site, and Cre recombinase. Heterozygote gene-targeted Tas1r1*^Cre^* mice were bred with Rosa26*^tdRFP^* mice [17], to generate Tas1r1*^Cre^*/Rosa26*^tdRFP^* mice. Tas1r1*^Cre^/*Rosa26*^tdRFP^* mice carry one recombinant *Tas1r1* and one recombinant *ROSA26* allele. The Cre recombinase is expressed under the control of the *Tas1r1* promoter; therefore a Cre-mediated recombination leads to excision of a transcriptional stop signal flanked by Lox-P sites and thus activates expression of tandem dimer red fluorescent protein (tdRFP) exclusively in Tas1r1 expressing cells. Tas2r131*^Cre^*/Rosa26*^tauGFP^* mice report the history of activity of the *Tas2r131* promoter. Briefly, Tas2r131*^Cre^* mouse line was generated by replacing the coding region of Tas2r131 by an expression cassette, containing the coding sequence of barley lectin, an internal ribosomal entry site, and Cre recombinase. Heterozygote gene-targeted Tas2r131*^Cre^* mice were bred with Rosa26*^tauGFP^* [18], mice to generate Tas2r131*^Cre^*/Rosa26*^tauGFP^* mouse line. These mice carry one recombinant *Tas2r131* and one recombinant *ROSA26* allele. Cre recombinase expression is controlled by *Tas2r131* promoter activity. Activation of *Tas2r131* promoter leads to the expression of Cre recombinase and therefore induces Cre-mediated recombination. This recombination event leads to the excision of a transcriptional stop signal flanked by Lox-P sites and thus activates expression of a green fluorescent protein (tauGFP) exclusively in Tas2r131 cells. So, the gene-targeted mouse lines (Tas1r1*^Cre^*/Rosa26*^tdRFP^*, Tas2r131*^Cre^*/Rosa26*^tauGFP^*) express both the Cre recombinase and the taste receptors in a heterozygote manner, as well as the fluorescent proteins tdRFP and tauGFP, respectively.

For studies on the effects of starvation on taste GPCR expression, C57BL/6J mice were deprived of food for 48 hours with *ad libitum* access to drinking water, as described previously [19].

Heart and tongue tissues were immediately dissected and either rapidly frozen in liquid nitrogen for RNA extraction or fixed with 4% paraformaldehyde for cryosectioning.

### Human Heart Tissue Samples

Following explantation from terminal heart failure patients undergoing heart transplantation, human hearts were immersed in preoxygenated (95% O_2_−5% CO_2_) modified Krebs’ solution containing (mmol/L) Na^+^125, K^+^5, Ca^2+^2.25, Mg^2+^0.5, Cl^−^ 98.5, SO4^2−^ 0.5, HCO_3_^−^ 29, HPO_4_^2−^ 1, and EDTA 0.04 [20], and samples of right atrial free wall and left ventricle were rapidly dissected and frozen in liquid nitrogen.

### Cardiomyocyte and Cardiac Fibroblast Isolation and Culture

Ventricular cardiomyocytes and fibroblasts were isolated, enzymatically digested and purified from one-day-old Sprague-Dawley rat pups, as described previously [21]. Briefly, neonates were killed by decapitation and the dissected ventricles were enzymatically digested with pancreatin and collagenase in a cell stirrer. Dissociated cardiac myocytes and fibroblasts were purified through a Percoll gradient to >99% homogeneity. Cardiomyocytes were plated on gelatin-coated tissue culture plates in Dulbecco’s modified Eagle’s medium (DMEM) supplemented with sodium bicarbonate, L-glutamine, essential and non-essential amino acids, vitamins, penicillin-streptomycin, amphotericin B, bromodeoxyuridine (BrDu) and 10% (v/v) new born calf serum. Myocytes were subsequently cultured in DMEM media supplemented with sodium bicarbonate, vitamins, essential and non-essential amino acids, sodium pyruvate, insulin, apo-transferrin, penicillin-streptomycin, amphotericin B, BrDu and 50 mmol/L KCl. Cardiac fibroblasts were cultured in DMEM supplemented with 10% (v/v) fetal bovine serum, sodium bicarbonate, penicillin-streptomycin and amphotericin B, and were grown to confluence in 6-well plates for RNA extraction. Nutrient deprivation experiments were performed for 24 hours on cultured myocytes in glucose-free DMEM media, supplemented as described above.

### RNA Isolation and Reverse Transcription Quantitative PCR (RT-qPCR)

Total RNA was extracted from human and rodent heart tissue at various time points, as well as from isolated cardiomyocytes and fibroblasts using TRIzol reagent (Life Technologies, Melbourne, VIC, Australia) following homogenization with a Polytron homogenizer, as per the manufacturer’s recommendations. RNA was DNase-treated to remove genomic DNA contaminants and then cDNA was synthesized from 1–6 µg of total RNA using Superscript III (Life Technologies, Melbourne, VIC, Australia) according to the manufacturer’s protocols. DNase treatment is particularly important for RT-qPCR experiments involving single exon genes, such as the TAS2Rs. Reactions without reverse transcriptase were performed in parallel to control for contamination with chromosomal DNA (Figure S1A and Table S3).

RT-qPCR was used to determine mRNA expression levels of cardiac GPCRs, taste GPCRs and taste-associated genes. 18S ribosomal RNA and *Gapdh* were used as stably expressed endogenous control genes for samples as indicated. mRNA expression levels were measured for all human and rat genes using Taqman probe chemistry with a StepOne Plus Real-Time PCR System or SYBR green chemistry on an ABI7000 instrument (Applied Biosystems, Melbourne, VIC, Australia). Primers and probes for human and rat GPCRs and taste-associated genes, 18S and *Gapdh* endogenous primers were from Applied Biosystems, while mouse taste receptor primers were from qPrimerDepot (accessible online: http://mouseprimerdepot.nci.nih.gov) [22]. All primer sets and part numbers, and primer sequences are shown in Table S1. Where possible, primers span exon boundaries, although this is not possible for the single exon Tas2/TAS2 genes. PCR products were run on native PAGE gels to confirm correct amplicon size (Figure S1B). For experiments using SYBR green chemistry, melt curve analysis was performed at the completion of each assay to determine the specificity of the PCR reaction. Expression of the gene of interest was normalized to *Gapdh* and 18S expression using the 2^−ΔΔCt^ method [23].

### Tissue Preparation and in situ Hybridization

Tissues were fixed in 4% paraformaldehyde overnight at 4°C, rinsed in phosphate-buffered saline and incubated in 30% sucrose solution for 6 hours. Following embedding and freezing in Tissue-Tek OCT (Sakura Finetek, Melbourne, VIC, Australia), 10 µm cryosections were collected on Superfrost (+) slides, and stored at −70°C. *In situ* hybridization was performed on rat heart using digoxigenin (DIG) labeled-cRNA probes as described previously [24]. See Table S2 for T3 and T7 RNA polymerase-containing primer sequences used for *in situ* probe synthesis. 10 µm cryosections were fixed, treated with proteinase K, washed in PBS, acetylated and hybridized with gene-specific probes overnight at 65°C. Sections were probed with anti-DIG antibody (Roche Diagnostics, Sydney, NSW, Australia) overnight at 4°C. Color was developed at room temperature using NBT/BCIP (Promega, Sydney, NSW, Australia).

### Data Analysis

Data expressed as mean±SEM. Statistical analyses were performed using one-way analysis of variance (ANOVA) with a Dunnett’s post-test or an unpaired student’s t-test, as indicated. P values below 0.05 were considered significant and are indicated as follows: *: P<0.05, **: P<0.01, ***P<0.001.

## Results

### Taste GPCRs are Expressed in the Rodent and Human Heart

**Figure 1:**
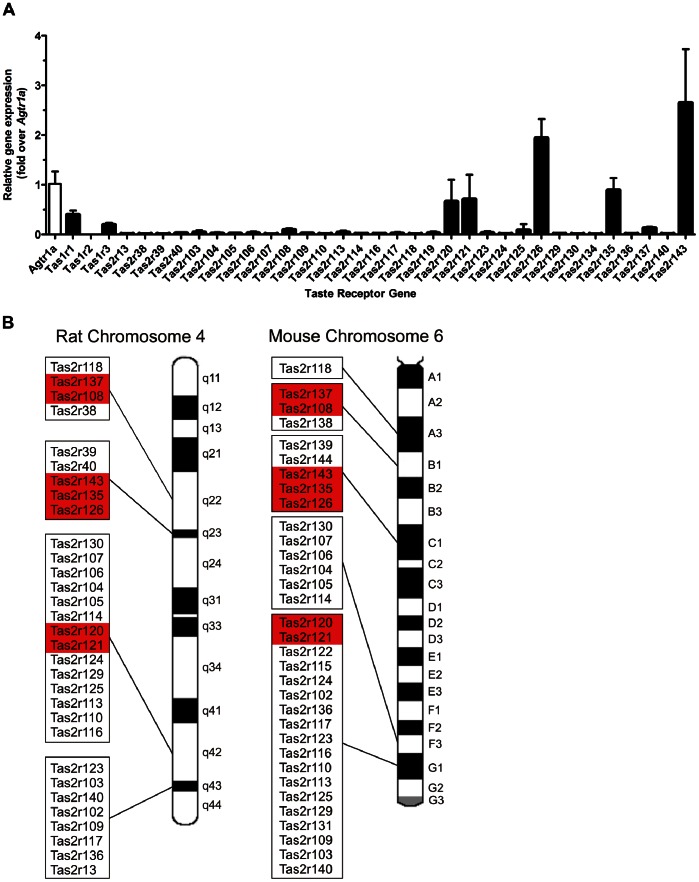
Taste GPCRs are expressed in the rodent heart. A RT-qPCR screen of taste GPCRs in neonatal rat whole heart (mean±SEM, n = 4, normalized for *Gapdh*, presented as fold change over the angiotensin II type 1a receptor (*Agtr1a*)). B Rodent *Tas2rs* are predominantly clustered on chromosome 4 (rat) and chromosome 6 (mouse). The taste GPCRs highlighted in red are detected in the heart, suggesting that these receptors may be under transcriptional regulation of a common regulatory element.

An RT-qPCR taste receptor screen in rat heart showed seven *Tas2* receptors (*Tas2r108*, *Tas2r120*, *Tas2r121*, *Tas2r126*, *Tas2r135*, *Tas2r137*, *Tas2r143*), as well as two members of the *Tas1* receptor family, *Tas1r1* and *Tas1r3*, were expressed in neonatal whole hearts, notably at levels comparable to another important cardiovascular GPCR, the angiotensin II type 1a receptor (*Agtr1a*)(Figure 1A). The seven cardiac-expressed *Tas2r*s do not display obvious sequence homology, however they cluster into three sub-groups of proximal receptors located within the larger group of *Tas2r*s on chromosome 4 in rat (or 6 in mouse), indicating possible common upstream regulatory elements (Figure 1B). Moreover, although the majority of rodent *Tas2r*s do not have corresponding human orthologs [25], each of the cardiac-expressed *Tas2* receptors described herein does, suggesting that the cardiac significance/function of these GPCRs may be evolutionarily conserved (Figure S2 and Table 1).

**Table 1:** Homology of taste GPCRs in human and rodents.

Human			Mouse			Rat
Gene symbol	Human vs. Mouse identity (%)		Gene symbol	Mouse vs. Rat identity (%)		Gene symbol
	Protein	DNA		Protein	DNA	
TAS1R1	74	79.6	Tas1r1	90.2	91.3	Tas1r1
TAS1R2	69.9	77.6	Tas1r2			Tas1r2
TAS1R3	73.6	75	Tas1r3	92.9	93.5	Tas1r3
TAS2R1	51.9	66.2	Tas2r119	85.3	89.7	Tas2r119
TAS2R3	64.2	77.8	Tas2r137	89.1	92.4	Tas2r137
TAS2R4	67	76.7	Tas2r108	88.2	91.1	Tas2r108
TAS2R5*						
TAS2R7	68.3	79.6	Tas2r130	93.3	92.3	Tas2r130
TAS2R8*						
TAS2R9*						
TAS2R10	57	71.2	Tas2r114	84.5	91.2	Tas2r114
TAS2R13	59	72.3	Tas2r121	82.6	88	Tas2r121
TAS2R14	48.9	66.6	Tas2r140	78.8	86.9	Tas2r140
TAS2R16	53.8	70.5	Tas2r118	92	93	Tas2r118
TAS2R19*						
TAS2R20	51.2	66.4	Tas2r120	82.7	88.8	Tas2r120
TAS2R30*						
TAS2R31*						
TAS2R38	65.6	76.5	Tas2r138	87.9	91.3	Tas2r38
TAS2R39	57.5	72.4	Tas2r139	85	89.6	Tas2r39
TAS2R40	66.4	78.5	Tas2r144	88.1	91.5	Tas2r40
TAS2R41	68.7	76.3	Tas2r126	90.9	92.6	Tas2r126
TAS2R42	50.5	66.4	Tas2r131	80	87.6	LOC100363053
TAS2R43*						
TAS2R45*						
TAS2R46*						
TAS2R50*						
TAS2R60	58.8	72	Tas2r135	93.1	94.1	Tas2r135
TAS2R62P		70.4	Tas2r143	87.4	91.7	Tas2r143
			Tas2r102	79.3	89	Tas2r13
			Tas2r103	76.6	86.4	Tas2r103
			Tas2r104	86.1	91.9	Tas2r104
			Tas2r105	83.9	88.7	Tas2r105
			Tas2r106	84.4	91	Tas2r106
			Tas2r107	85.3	89.6	Tas2r107
			Tas2r109	75	85.2	Tas2r109
			Tas2r110	77.3	85.6	Tas2r110
			Tas2r113	79	88.3	Tas2r113
			Tas2r116	73.4	85	Tas2r116
			Tas2r117	77	87.8	Tas2r117
			Tas2r123	80.4	87	Tas2r123
			Tas2r124	81.6	88.3	Tas2r124
			Tas2r125	76.1	85.6	Tas2r125
			Tas2r129	78.1	87	Tas2r129
			Tas2r134	82	88.7	Tas2r134
			Tas2r136	75.5	85.4	Tas2r136

Human taste receptors are shown on the left, with the corresponding mouse and rat taste receptor and percentage identity at the protein and DNA level, taken from the NCBI database (http://www.ncbi.nlm.nih.gov/homologene). Where human taste receptor homologs do exist, they are shown aligned to their rodent counterparts, with protein and DNA identity to mouse and rat sequences. Human specific taste GPCRs are denoted with an asterisk (*).

**Figure 2:**
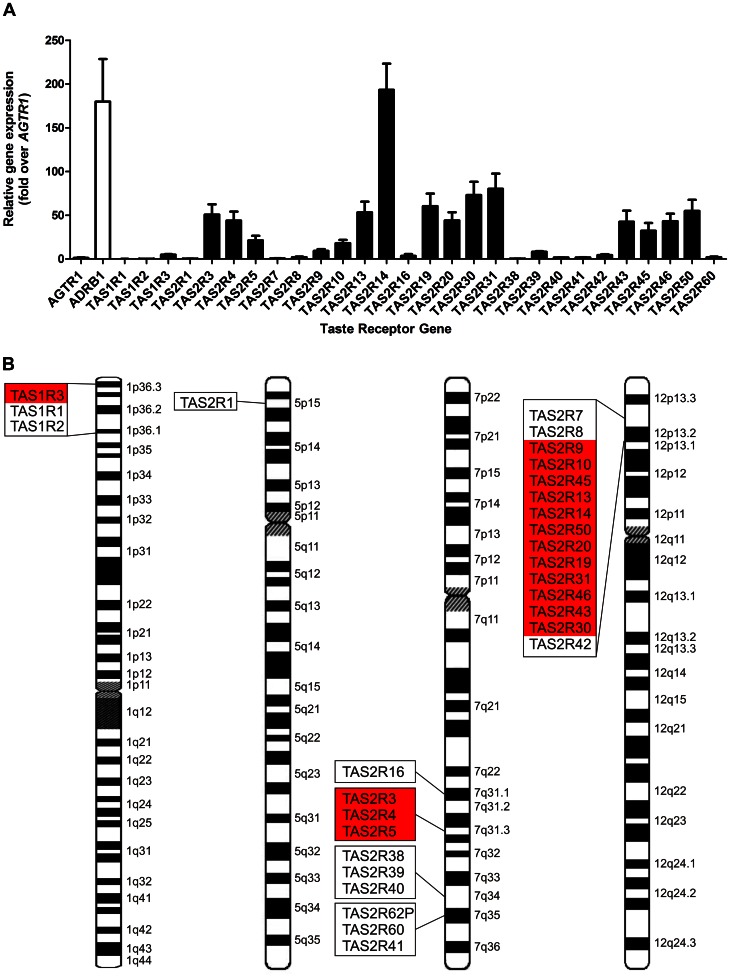
Taste GPCRs are expressed in the human heart. **A** RT-qPCR screen of taste GPCRs in human left ventricle (mean±SEM, n = 5, normalized for 18S, presented as fold change over the angiotensin II type 1 receptor (*AGTR1*)). The abundantly expressed β_1_-adrenergic receptor (*ADRB1*) is shown as a comparator. **B** Human *TASRs* are localized on chromosomes 1, 5, 7 and 12, with the majority of TAS2Rs expressed in the heart (highlighted in red) present in a cluster on chromosome 12.

To begin to empirically test this possibility, we obtained heart tissue from failing human hearts and performed an RT-qPCR screen for the taste GPCRs (Fig. 2A). Transcripts for more than half of the *TAS2R* family were detected in these human left ventricle samples, at comparatively high levels relative to the angiotensin II type 1 receptor (*AGTR1*). Remarkably, *TAS2R14* is expressed at comparable levels to the β_1_-adrenergic receptor (*ADRB1*), an abundantly expressed GPCR that is a critical mediator of chronotropy and ionotropy in the heart. The same receptors and relative expression patterns were observed for right atrial tissue (Figure S3). It is also interesting to note that the human *TAS2R*s detected in heart are clustered in close genomic proximity on chromosomes 7 and 12. As in rodents, this genomic organization seems to correlate with the expression of particular subsets of *TAS2R*s in the heart (Figure 2B).

### Localization of Taste GPCRs in the Rodent Heart

**Figure 3:**
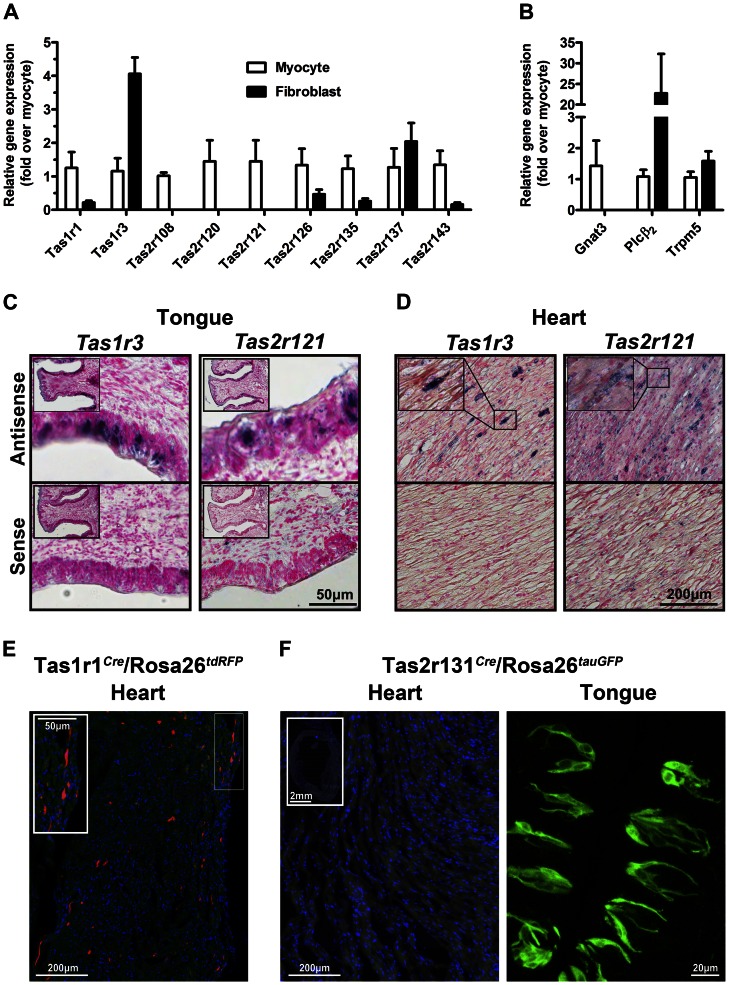
Taste GPCRs are localized in isolated cardiac cells. **A** Taste receptors were detected in cultured neonatal cardiomyocytes and fibroblasts by RT-qPCR (mean±SEM, n = 4, normalized for *Gapdh* and expressed relative to myocytes). **B** The taste receptor signal transduction genes, including G protein (*Gnat3*), second messenger (*PLCβ2*) and channel (*Trpm5*) are also expressed in myocytes and fibroblasts (mean±SEM, n = 4, normalized for *Gapdh* and expressed relative to myocytes). **C** *In situ* hybridization using digoxigenin-labeled cRNA probes specific for *Tas1r3* (left panels) and *Tas2r121* (right panels) show expression in the taste buds of the circumvallate papillae (inset shows low magnification) and **D** in heart tissue (inset shows higher magnification of boxed region). Specific labeling (blue/black) is observed using antisense probes, but not sense probes. **E** *Tas1r1* is expressed in mouse ventricular tissue from Tas1r1*^Cre^*/Rosa26*^tdRFP^* mice, where red fluorescent cells report activity of the *Tas1r1* promoter. **F** In contrast, in heart sections from the Tas2r131*^Cre^*/Rosa26*^tauGFP^* reporter mouse line, there are no fluorescently-labeled myocytes (inset shows low magnification view of heart). *Tas2r131*–expressing cells are clearly labeled in the circumvallate papillae as control. Scale bars are as indicated.

To further characterize the taste GPCRs that were identified in our initial RT-qPCR screen, we investigated their localization in cardiac cells. Several taste GPCRs were readily detected in primary cultures of both isolated, purified neonatal rat cardiac cells, and with the exception of *Tas1r3*, were enriched in cardiomyocytes over fibroblasts (Figure 3A). Several genes commonly implicated in the taste receptor signaling cascade, specifically *Gnat3*, *Plcβ2* and *Trpm5*, were also expressed in cardiomyocytes (Figure 3B).

Given the lack of selective and specific antibodies for the taste GPCRs, *in situ* hybridization was used to localize taste receptor expression in heart. In representative adult heart sections, *Tas1* (*Tas1r3*) and *Tas2* (*Tas2r121*) receptors showed distinct labeling in a small proportion of cardiac cells distributed throughout the myocardium (Figure 3C and D and inset). The probes labeled taste receptor-expressing cells in the circumvallate papillae of the tongue, whereas no staining was visible in sense controls. The expression of *Tas1r1* in discrete cardiac cells within the myocardium was also confirmed in a *Tas1r1* gene-targeted mouse (Tas1r1*^Cre^*/Rosa26*^tdRFP^*)(Figure 3E). Importantly, another gene-targeted mouse, Tas2r131*^Cre^*/Rosa26*^tauGFP^* exhibited strong labeling of taste receptor-expressing cells in the circumvallate papillae, but no cardiac expression, consistent with our RT-qPCR data (Figure 3F). Thus, specific taste receptor genes are expressed in cardiac cells.

### Taste GPCRs are Developmentally Regulated in the Heart

**Figure 4:**
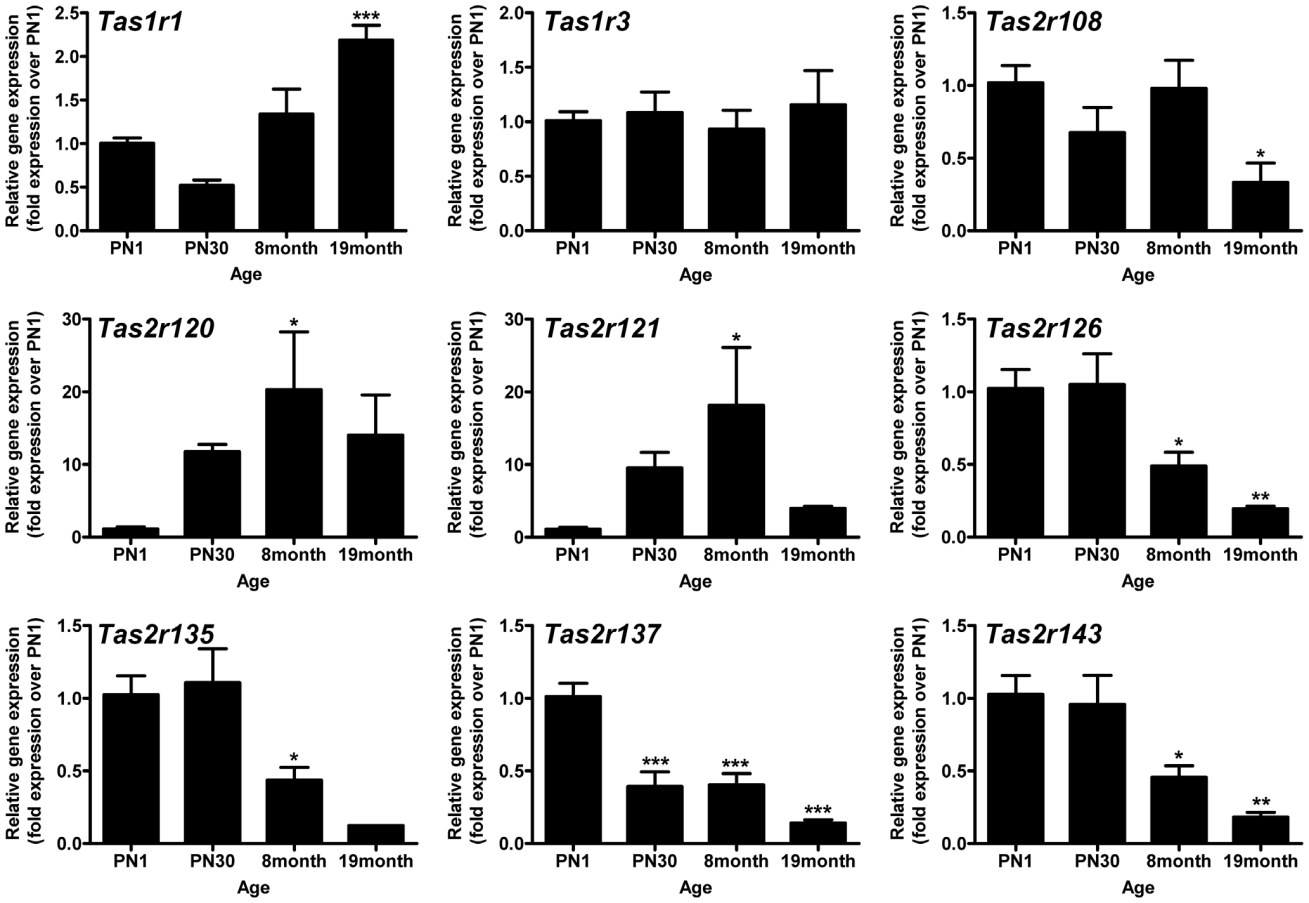
Taste GPCRs are regulated in the heart during postnatal life *in vivo*. Taste GPCR mRNA expression levels at different postnatal ages from postnatal day 1 (PN1) to 19 months. Gene expression levels are normalized to *Gapdh* and expressed relative to PN1 (mean±SEM, expressed relative to PN1, n = 4, *P<0.05; **P<0.01, ***P<0.001, one-way ANOVA with a Dunnett’s post-test).

We characterized the ontogeny of taste receptor expression in the heart, at multiple time points from neonatal (postnatal day 1) to 19 months of age (Figure 4). *Tas1r1* expression increased 2-fold in senescent rat hearts, whereas *Tas1r3* expression levels remained constant throughout life. The *Tas2rs* displayed contrasting temporal patterns of expression. *Tas2r120* and *Tas2r121* expression increased nearly 20-fold in adult rats relative to neonate hearts. The remaining cardiac-expressed *Tas2rs* (108, 126, 135, 137 and 143) decreased in abundance with age (3–8 fold at 19 months).

### Taste GPCRs are Regulated under Conditions of Starvation Both in vitro and in vivo

**Figure 5:**
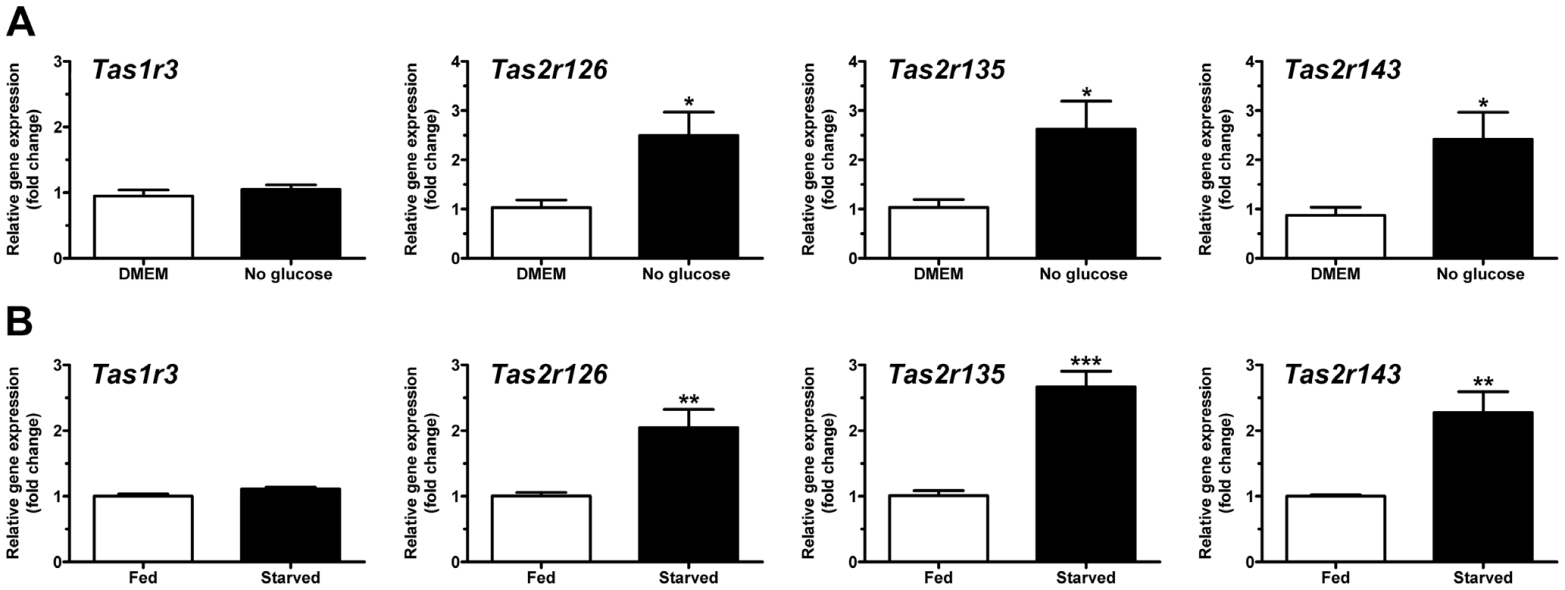
Taste GPCRs are regulated in the heart under conditions of starvation both *in vitro* and *in vivo*. **A** *Tas2r126*, *Tas2r135* and *Tas2r143* mRNA expression levels in cultured neonatal rat ventricular myocytes deprived of glucose for 24 h (mean±SEM, n = 4, fold change over glucose-containing myocyte media conditions (DMEM)). **B** *Tas2r126*, *Tas2r135* and *Tas2r143* mRNA expression levels in hearts of mice starved for 48 h (mean±SEM, n = 4, fold change over fed (*ad lib*) controls, *P<0.05; **P<0.01, ***P<0.001, unpaired student’s t test).

Given the purported role of *Tas1* GPCRs in nutrient sensing, we investigated the effect of nutrient deprivation on expression of taste receptors in cultured rat cardiomyocytes. Contrary to our expectations, *Tas1r1* and *Tas1r3* expression levels were unchanged in myocytes following 24 hours glucose deprivation. Similarly, there was no effect of amino acid deprivation on taste GPCR expression (Figure S4). The regulation of *Tas2* GPCRs has not been thoroughly investigated with respect to nutrient status. Intriguingly, the expression of *Tas2r126*, *Tas2r135* and *Tas2r143* increased 2–3 fold in the absence of glucose, whereas the remaining taste GPCRs expressed were unchanged (Figure 5A, Figure S5). These data were verified *in vivo* in a mouse model of starvation, where the same *Tas2rs* were upregulated after 48 hours food deprivation (Figure 5B). Taken together, these data suggest that the cardiac expression of *Tas2* receptors could have physiological relevance as novel metabolic/nutrient sensors in the heart.

## Discussion

Using RT-qPCR, *in situ* hybridization, and gene-targeted reporter mice, we demonstrate, for the first time, the expression of individual *Tas1* and *Tas2* receptors in cardiomyocytes, fibroblasts and heart tissue. In rodents, these receptors are expressed throughout the myocardium in a small subset of cardiac cells and some receptors are upregulated at the transcript level *in vitro* and *in vivo* upon nutrient starvation. The cardiac-expressed *Tas2r*s are located in genomic clusters and share developmental and physiological expression patterns, suggesting common regulatory control. Each of these receptors described has a human homolog, implying conservation across eutheria. Indeed, in human heart tissue samples obtained from heart transplant patients, taste GPCR transcripts are readily detected, most strikingly the *TAS2R*s. The delineation of *Tas1/Tas2* GPCRs in heart opens up new avenues of cardiovascular research.

Recent large-scale efforts to profile and characterize GPCR expression in different tissues, demonstrate that the heart expresses more than 100 GPCRs [3], [4], [26], [27], although these studies have pragmatically excluded the chemosensory receptors. Collectively, these studies raise the possibility that we are overlooking important physiological and pathophysiological GPCR targets – specifically by focusing only on well characterized GPCRs with available, potent and selective ligands. Recent work by Insel and colleagues has epitomized this idea in demonstrating that a previously unheralded, albeit ubiquitously expressed GPCR (protease activated receptor 1, PAR1), may play an important role in cardiac fibrosis [28]. Our study is the first to systematically describe taste GPCRs in heart and supports the idea that previous studies may have been too narrow in their scope in profiling GPCR expression across tissues.

The type 1 *(TAS1/Tas1)* family taste receptors have a nutrient sensing role in the tongue, as well as other tissues, including the gastrointestinal tract and the brain [5], [29], [30]. Our specific identification in heart of *Tas1r1*/*Tas1r3* is consistent with a nutrient-sensing role and may indicate a role in metabolic regulation. Indeed, Tas1r1/Tas1r3 have very recently been implicated as direct upstream amino acid sensors for the mTORC1-mediated autophagy pathway [9]. While not entirely surprising, given their well-described roles as broadly-tuned amino acid sensors, this newly attributed function to Tas1rs outside the mouth (and our discovery of them in heart) reinforces the notion that these GPCRs are involved in far more than taste.

Another major finding in our study is the prevalence of the type 2 “bitter” (*TAS2/Tas2)* GPCRs in both human and rodent hearts. We also detected the genes commonly associated with taste GPCR signaling, endowing the heart with taste-receptor like signaling potential, although there is no *a priori* reason to assume that these are the cardiac effectors of Tas2 activation. The endogenous complement of proteins in the heart would likely dictate the signal transduction and hence cardiac function of these GPCRs, although the downstream components remain to be delineated. While Tas2rs are predominantly located within multigene clusters in mouse, rat and human genomes, there is little known about their tissue-specific and transcriptional regulation [31]. Nevertheless, the proximal chromosomal organization and clustering of the subset of cardiac-expressed taste GPCRs, as well as their shared regulatory patterns, suggest possible transcriptional co-regulation.

What could be the potential function for a TAS2 receptor in the heart? The localization of taste GPCRs in discrete heart cells parallels that seen in the respiratory and gastrointestinal systems [13], [15], suggesting that these may function as specialized cells that sense the extracellular environment and influence surrounding tissue. In this regard, the upregulation of a subset of the *Tas2* GPCRs in the heart following nutrient deprivation and starvation is interesting. These findings could reflect a potential function as nutrient sensors in heart. Interestingly, taste receptors are upregulated in several conditions where specific nutrients are depleted [5], [32], and a single nucleotide polymorphism in a human TAS2R has been linked to deficits in glucose homeostasis [33]. Similarly, gene variants in TAS2Rs are related to disease – a polymorphism in TAS2R38 contributes to an increased susceptibility to respiratory infection [34] and a mutation in TAS2R50 has been associated with cardiovascular disease in several population-based prospective studies [35], [36], but the mechanisms by which the variants of these TAS2 genes influence the pathophysiology of disease are mostly unknown. Although the present findings do not discriminate between increased TAS2 expression in a subpopulation of cells versus an increase in the number of cells expressing the receptor, the cardiac expression of taste GPCRs raises the possibility of a direct effect, beyond oral sensation [37].

An obvious consideration for the cardiac expression of Tas2rs is their ligand-mediated activation – from where do these ligands originate and how would they get to the heart? At present, the best candidates for activating TAS2Rs outside the gustatory system are inhaled irritants and toxins [15], [34]. It is possible that exogenous toxins could be taken into the circulatory system to target the cardiac-Tas2rs. An alternative possibility is that there are endogenous TAS2R agonists produced within the circulation and/or cardiovascular tissues. Indeed, there is a growing literature pertaining to the metabolite ligands for GPCRs in physiology and disease (recently reviewed in [38], [39]). Notably, metabolite ligands have been associated with a variety of receptor systems and may have potential therapeutic applications in a variety of metabolic diseases and in pain [40], [41], [42], [43], [44].

Clearly, the identification of TAS2Rs in the heart warrants further investigation in cardiac physiology and disease. We are currently working to identify potent and selective ligands for these *Tas2rs* in rodents, which will appreciably aid efforts towards their functional characterization in heart. In addition, given the interesting species similarities/differences in TAS2Rs, it will be important to also direct future studies to cells and tissues derived from humans, where the characterization of TAS2R ligand binding profiles [10] and pharmacological tools (e.g. small molecule TAS2R antagonists [45]) are slightly more advanced. Indeed, while chemosensory receptors represent the majority of GPCRs in our genome, we are yet to fully understand the extent of their biological importance in tissues beyond the nose and mouth. We agree with Munger and colleagues that the extraoral expression of TAS2Rs may be a potential confounder in the application and understanding of GPCR-based therapeutics [46], but reiterate that they could also be possible drug targets in their own right.

## Supporting Information

Figure S1 A Representative RT-qPCR amplification plot for rat *Tas2r143* in the presence (black traces) or absence (blue traces) of reverse transcriptase. As shown in Table S3, four independent samples of neonatal rat heart mRNA were assayed in triplicate. All +RT samples amplified with Ct values approximating 27.4, whereas 9 of 12 of the –RT replicates failed to amplify. The remaining 3 replicates amplified at an average CT value of 34.8, generally indistinguishable from background. **B** The correct amplicon sizes of cardiac-expressed TasRs were confirmed by running RT-qPCR samples (+ and – RT) on a 12% native PAGE gel. Shown are three representative rat Tas2rs (*Tas2r126*, *Tas2r135* and *Tas2r143*) running at their expected molecular size (139 base pairs, 159 base pairs and 97 base pairs, respectively) relative to marker bands at 300, 150 and 50 base pairs, as indicated.
(TIF)

Figure S2 Schematic showing the genomic organization of the *Tas2r143*, *135* and *126* cluster of taste GPCRs in mouse, and the mammalian conservation. Generated using the UCSC Genome Browser database: http://genome.ucsc.edu/.
(TIF)

Figure S3 Taste GPCRs are expressed in the human right atria. RT-qPCR screen of taste GPCRs in human right atria (mean ± SEM, n = 5, normalized for 18S, presented as fold change over the angiotensin II type 1 receptor (*AGTR1*)). The abundantly expressed β_1_-adrenergic receptor (*ADRB1*) is shown as a comparator.
(TIF)

Figure S4 Amino acid deprivation (24 h) has no effect on *Tas1* or *Tas2* GPCR mRNA expression in cultured neonatal rat ventricular myocytes. Data expressed as mean±SEM, n = 4, fold change over amino acid-containing myocyte media conditions (DMEM).
(TIF)

Figure S5 Glucose deprivation (24 h) does not modulate the mRNA expression of a subset of taste GPCRs in cultured neonatal rat ventricular myocytes. Data expressed as mean±SEM, n = 4, fold change over glucose-containing myocyte media conditions (DMEM).
(TIF)

Table S1 RT-qPCR primers and probes and their sequences/part numbers.
(DOCX)

Table S2 *In situ* hybridization probe primer sequences, including T7 and T3 RNA polymerase binding sites.
(DOCX)

Table S3 RT-qPCR experiments were performed in the presence and absence of reverse transcriptase following DNase treatment to confirm the specific amplification of cDNA, in contrast to genomic DNA.
(DOCX)
