# Supplementary figures and images for: Expression, Regulation and Putative Nutrient-Sensing Function of Taste GPCRs in the Heart
Source: PLoS One. 2013 May 15;8(5):e64579. doi: 10.1371/journal.pone.0064579 (PMC3655793; doi:10.1371/journal.pone.0064579)

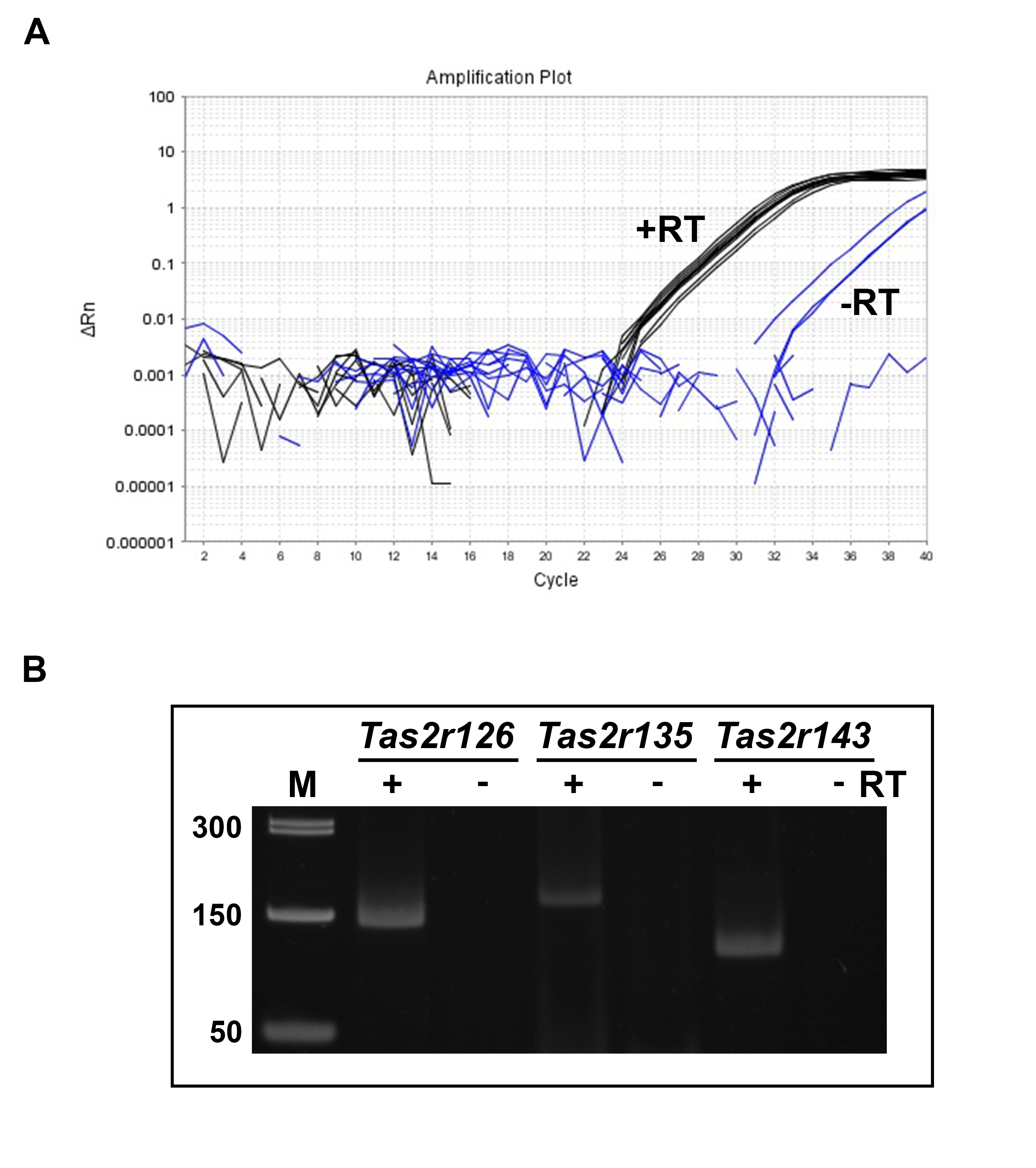

Supplement: Figure S1 — A Representative RT-qPCR amplification plot for rat Tas2r143 in the presence (black traces) or absence (blue traces) of reverse transcriptase. As shown in Table S3, four independent samples of neonatal rat heart mRNA were assayed in triplicate. All +RT samples amplified with Ct values approximating 27.4, whereas 9 of 12 of the –RT replicates failed to amplify. The remaining 3 replicates amplified at an average CT value of 34.8, generally indistinguishable from background. B The correct amplicon sizes of cardiac-expressed TasRs were confirmed by running RT-qPCR samples (+ and – RT) on a 12% native PAGE gel. Shown are three representative rat Tas2rs (Tas2r126, Tas2r135 and Tas2r143) running at their expected molecular size (139 base pairs, 159 base pairs and 97 base pairs, respectively) relative to marker bands at 300, 150 and 50 base pairs, as indicated. (TIF) [file pone.0064579.s001.tif]

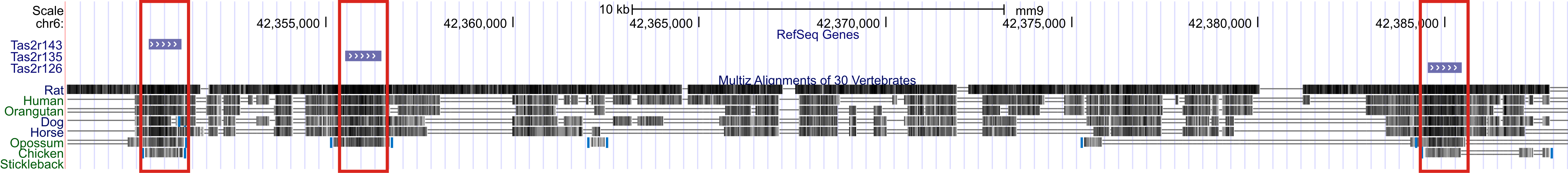

Supplement: Figure S2 — Schematic showing the genomic organization of the Tas2r143, 135 and 126 cluster of taste GPCRs in mouse, and the mammalian conservation. Generated using the UCSC Genome Browser database: http://genome.ucsc.edu/. (TIF) [file pone.0064579.s002.tif]

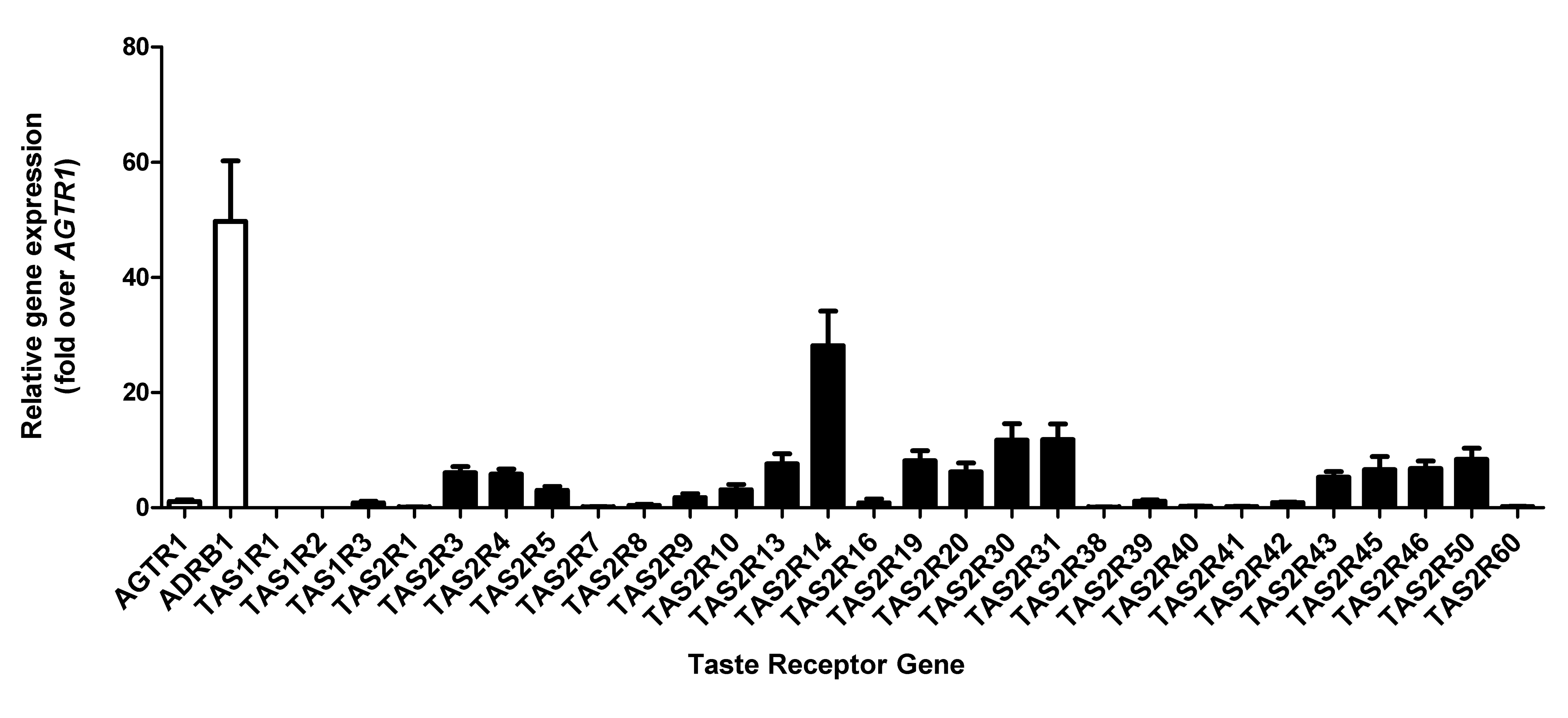

Supplement: Figure S3 — Taste GPCRs are expressed in the human right atria. RT-qPCR screen of taste GPCRs in human right atria (mean ± SEM, n = 5, normalized for 18S, presented as fold change over the angiotensin II type 1 receptor (AGTR1)). The abundantly expressed β1-adrenergic receptor (ADRB1) is shown as a comparator. (TIF) [file pone.0064579.s003.tif]

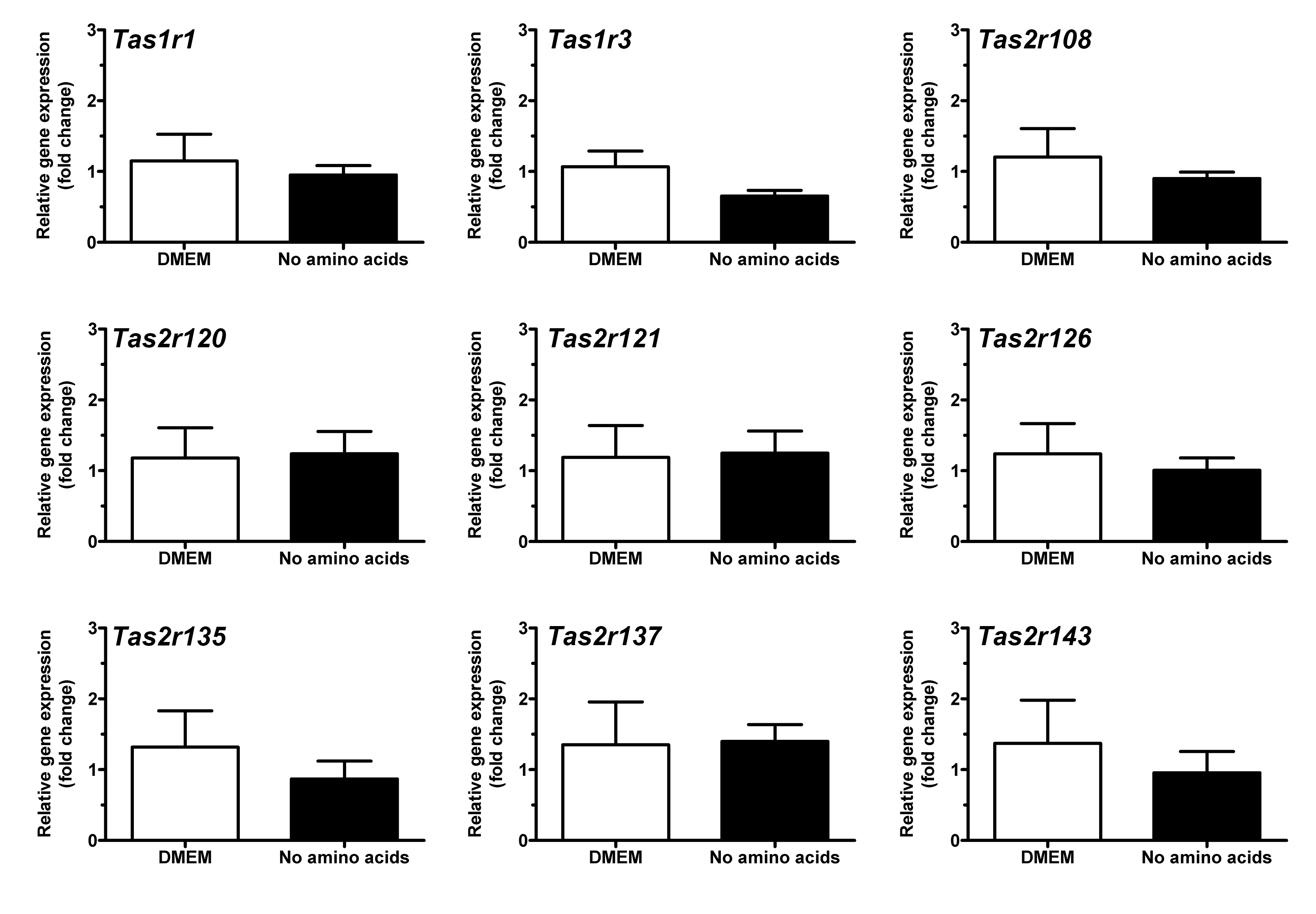

Supplement: Figure S4 — Amino acid deprivation (24 h) has no effect on Tas1 or Tas2 GPCR mRNA expression in cultured neonatal rat ventricular myocytes. Data expressed as mean±SEM, n = 4, fold change over amino acid-containing myocyte media conditions (DMEM). (TIF) [file pone.0064579.s004.tif]

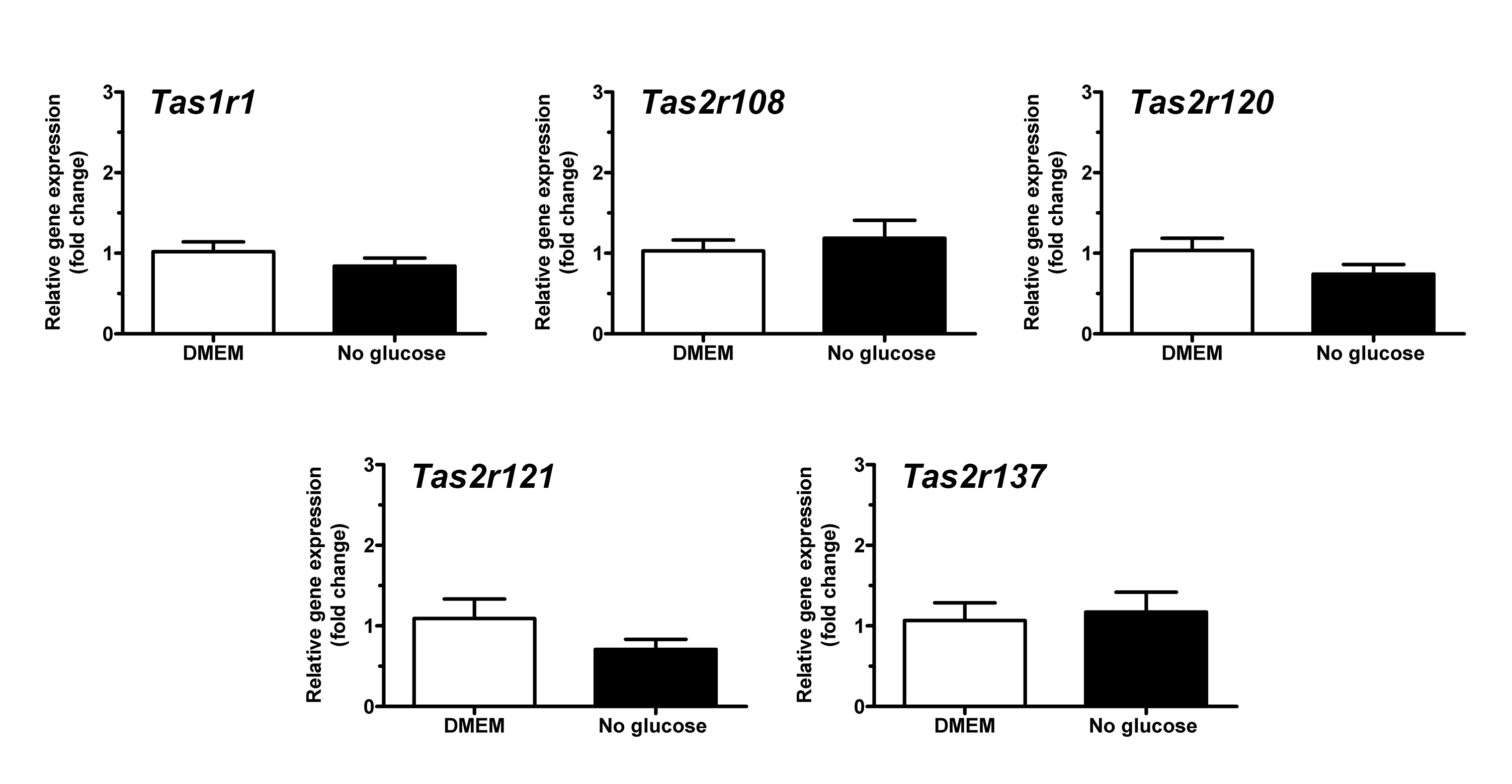

Supplement: Figure S5 — Glucose deprivation (24 h) does not modulate the mRNA expression of a subset of taste GPCRs in cultured neonatal rat ventricular myocytes. Data expressed as mean±SEM, n = 4, fold change over glucose-containing myocyte media conditions (DMEM). (TIF) [file pone.0064579.s005.tif]
