# Supplementary material for: Expression, Regulation and Putative Nutrient-Sensing Function of Taste GPCRs in the Heart
Source: PLoS One. 2013 May 15;8(5):e64579. doi: 10.1371/journal.pone.0064579 (PMC3655793; doi:10.1371/journal.pone.0064579)
Supplement: Table S1 — RT-qPCR primers and probes and their sequences/part numbers. (DOCX) [file pone.0064579.s006.docx]

**Table S1: RT-qPCR primers and probes and their sequences/part numbers**

| Gene symbol | Gene name | Reference sequence | Source | Sequence / Part number |
| --- | --- | --- | --- | --- |
| Agtr1a | Angiotensin II receptor, type 1a | NM_030985.4 | Applied Biosystems | Rn02758772_s1 |
| Gnat3 | Guanine nucleotide binding protein, alpha transducing 3 | NM_173139.1 | Applied Biosystems | Rn00597619_m1 |
| Plcb2 | Phospholipase C, beta 2 | NM_053478.1 | Applied Biosystems | Rn00585063_m1 |
| Trpm5 | Transient receptor potential cation channel, subfamily M, member 5 | NM_001191896.1 | Applied Biosystems | Rn01479552_m1 |
| Tas1r1 | Taste receptor, type 1, member 1 | NM_053305.1 | Applied Biosystems | Rn01516038_m1 |
| Tas1r2 | Taste receptor, type 1, member 2 | Q9Z0R7.1 | Applied Biosystems | Rn01515494_m1 |
| Tas1r3 | Taste receptor, type 1, member 3 | NM_130818.1 | Applied Biosystems | Rn00590759_g1 |
| Tas2r13 | Taste receptor, type 2, member 13 | NM_023998.1 | Applied Biosystems | Rn00821169_g1 |
| Tas2r38 | Taste receptor, type 2, member 38 | NM_001024685.1 | Applied Biosystems | Rn02396417_s1 |
| Tas2r39 | Taste receptor, type 2, member 39 | NM_001080905.1 | Applied Biosystems | Rn04218919_s1 |
| Tas2r40 | Taste receptor, type 2, member 40 | NM_001025150.1 | Applied Biosystems | Rn02585844_s1 |
| Tas2r103 | Taste receptor, type 2, member 103 | NM_001166690.1 | Applied Biosystems | Rn04181206_s1 |
| Tas2r104 | Taste receptor, type 2, member 104 | NM_001166681.1 | Applied Biosystems | Rn04181182_s1 |
| Tas2r105 | Taste receptor, type 2, member 105 | NM_023999.1 | Applied Biosystems | Rn00577003_s1 |
| Tas2r106 | Taste receptor, type 2, member 106 | NM_001166680.1 | Applied Biosystems | Rn02396427_s1 |
| Tas2r107 | Taste receptor, type 2, member 107 | NM_023995.1 | Applied Biosystems | Rn04181173_s1 |
| Tas2r108 | Taste receptor, type 2, member 108 | NM_001024686.1 | Applied Biosystems | Rn00576969_s1 |
| Tas2r109 | Taste receptor, type 2, member 109 | NM_001080939.1 | Applied Biosystems | Rn03415903_s1 |
| Tas2r110 | Taste receptor, type 2, member 110 | NM_001166677.1 | Applied Biosystems | Rn01493128_sH |
| Tas2r113 | Taste receptor, type 2, member 113 | NM_001166689.1 | Applied Biosystems | Rn04181200_s1 |
| Tas2r114 | Taste receptor, type 2, member 114 | NM_023996.1 | Applied Biosystems | Rn00576975_s1 |
| Tas2r116 | Taste receptor, type 2, member 116 | NM_001166679.1 | Applied Biosystems | Rn01462692_s1 |
| Tas2r117 | Taste receptor, type 2, member 117 | NM_001166682.1 | Applied Biosystems | Rn04181184_s1 |
| Tas2r118 | Taste receptor, type 2, member 118 | NM_023994.1 | Applied Biosystems | Rn00576960_s1 |
| Tas2r119 | Taste receptor, type 2, member 119 | NM_023993.1 | Applied Biosystems | Rn00576950_s1 |
| Tas2r120 | Taste receptor, type 2, member 120 | NM_001080937.1 | Applied Biosystems | Rn03415897_s1 |
| Tas2r121 | Taste receptor, type 2, member 121 | NM_023997.1 | Applied Biosystems | Rn00576987_s1 |
| Tas2r123 | Taste receptor, type 2, member 123 | NM_173336.1 | Applied Biosystems | Rn00756563_s1 |
| Tas2r124 | Taste receptor, type 2, member 124 | NM_001080938.1 | Applied Biosystems | Rn01492457_g1 |
| Tas2r125 | Taste receptor, type 2, member 125 | NM_001109596.1 | Applied Biosystems | Rn01459591_s1 |
| Tas2r126 | Taste receptor, type 2, member 126 | NM_139335.1 | Applied Biosystems | Rn00595098_s1 |
| Tas2r129 | Taste receptor, type 2, member 129 | NM_001166683.1 | Applied Biosystems | Rn01459135_s1 |
| Tas2r130 | Taste receptor, type 2, member 130 | XM_001074122.1 | Applied Biosystems | Rn01459742_s1 |
| Tas2r134 | Taste receptor, type 2, member 134 | NM_001013915.1 | Applied Biosystems | Rn02395456_s1 |
| Tas2r135 | Taste receptor, type 2, member 135 | NM_001025062.1 | Applied Biosystems | Rn02585807_s1 |
| Tas2r136 | Taste receptor, type 2, member 136 | NM_001166678.1 | Applied Biosystems | Rn01749087_s1 |
| Tas2r137 | Taste receptor, type 2, member 137 | NM_001025149.1 | Applied Biosystems | Rn01500928_s1 |
| Tas2r140 | Taste receptor, type 2, member 140 | NM_001085397.1 | Applied Biosystems | Rn01492598_s1 |
| Tas2r143 | Taste receptor, type 2, member 143 | NM_001025061.1 | Applied Biosystems | Rn02585801_s1 |
| AGTR1 | Angiotensin II receptor, type 1 | NM_031850.3 | Applied Biosystems | Hs00258938_m1 |
| ADRB1 | Adrenoceptor beta 1 | NM_000684.2 | Applied Biosystems | Hs02330048_s1 |
| TAS1R1 | Taste receptor, type 1, member 1 | NM_138697.3 | Applied Biosystems | Hs00602668_m1 |
| TAS1R2 | Taste receptor, type 1, member 2 | NM_152232.2 | Applied Biosystems | Hs01027711_m1 |
| TAS1R3 | Taste receptor, type 1, member 3 | NM_152228.1 | Applied Biosystems | Hs01026531_g1 |
| TAS2R1 | Taste receptor, type 2, member 1 | NM_019599.2 | Applied Biosystems | Hs00251930_s1 |
| TAS2R3 | Taste receptor, type 2, member 3 | NM_016943.2 | Applied Biosystems | Hs00249942_s1 |
| TAS2R4 | Taste receptor, type 2, member 4 | NM_016944.1 | Applied Biosystems | Hs00249946_s1 |
| TAS2R5 | Taste receptor, type 2, member 5 | NM_018980.2 | Applied Biosystems | Hs01549633_s1 |
| TAS2R7 | Taste receptor, type 2, member 7 | NM_023919.2 | Applied Biosystems | Hs00256778_s1 |
| TAS2R8 | Taste receptor, type 2, member 8 | NM_023918.1 | Applied Biosystems | Hs00256766_s1 |
| TAS2R9 | Taste receptor, type 2, member 9 | NM_023917.2 | Applied Biosystems | Hs00256757_s1 |
| TAS2R10 | Taste receptor, type 2, member 10 | NM_023921.1 | Applied Biosystems | Hs00256794_s1 |
| TAS2R13 | Taste receptor, type 2, member 13 | NM_023920.2 | Applied Biosystems | Hs01059805_s1 |
| TAS2R14 | Taste receptor, type 2, member 14 | NM_023922.1 | Applied Biosystems | Hs00256800_s1 |
| TAS2R16 | Taste receptor, type 2, member 16 | NM_016945.2 | Applied Biosystems | Hs00249955_s1 |
| TAS2R20 | Taste receptor, type 2, member 20 | NM_176889.2 | Applied Biosystems | Hs00604340_s1 |
| TAS2R31 | Taste receptor, type 2, member 31 | NM_176885.2 | Applied Biosystems | Hs00604313_sH |
| TAS2R38 | Taste receptor, type 2, member 38 | NM_176817.4 | Applied Biosystems | Hs00604294_s1 |
| TAS2R39 | Taste receptor, type 2, member 39 | NM_176881.2 | Applied Biosystems | Hs00603443_s1 |
| TAS2R40 | Taste receptor, type 2, member 40 | NM_176882.1 | Applied Biosystems | Hs00602589_s1 |
| TAS2R41 | Taste receptor, type 2, member 41 | NM_176883.2 | Applied Biosystems | Hs00603461_s1 |
| TAS2R42 | Taste receptor, type 2, member 42 | NM_181429.1 | Applied Biosystems | Hs00704057_s1 |
| TAS2R43 | Taste receptor, type 2, member 43 | NM_176884.2 | Applied Biosystems | Hs00853105_sH |
| TAS2R45 | Taste receptor, type 2, member 45 | NM_176886.1 | Applied Biosystems | Hs00820227_s1 |
| TAS2R46 | Taste receptor, type 2, member 46 | NM_176887.2 | Applied Biosystems | Hs00853124_s1 |
| TAS2R30 | Taste receptor, type 2, member 30 | NM_001097643.1 | Applied Biosystems | Hs03054740_sH |
| TAS2R19 | Taste receptor, type 2, member 19 | NM_176888.1 | Applied Biosystems | Hs00853130_s1 |
| TAS2R50 | Taste receptor, type 2, member 50 | NM_176890.2 | Applied Biosystems | Hs00604351_s1 |
| TAS2R60 | Taste receptor, type 2, member 60 | NM_177437.1 | Applied Biosystems | Hs00603474_s1 |
| 18S | 18S ribosomal RNA |  | Applied Biosystems | 4308329 |
| Gapdh | Glyceraldehyde-3-phosphate  dehydrogenase |  | Applied Biosystems | 4308313 |
| Tas1r1 | Taste receptor, type 1, member 1 | NM_031867.2 | qPrimerDepot | F: GTTCTCCCTCCATGCTGACT |
|  |  |  |  | R: CATGGCTTGGAAGAGGTGAT |
| Tas1r3 | Taste receptor, type 1, member 3 | NM_031872.2 | qPrimerDepot | F: TGCTATGACTGCGTGGACTG |
|  |  |  |  | R: CTGTGCTTTTCTCTGGGGAC |
| Tas2r108 | Taste receptor, type 2, member 108 | NM_020502.1 | qPrimerDepot | F: TATTTGTGTTTGCTGCCTCG |
|  |  |  |  | R: AATTCTGCGACTGTTGACCC |
| Tas2r121 | Taste receptor, type 2, member 121 | NM_207024.1 | qPrimerDepot | F: CCTGGTCTCCTTCATCCTGT |
|  |  |  |  | R: TTAGTGCTGGGGTCTCGTTC |
| Tas2r126 | Taste receptor, type 2, member 126 | NM_207028.1 | qPrimerDepot | F: TGGTTGAAGTGGAGATTCCC |
|  |  |  |  | R: TGGTTTCCCCAAAAGAACAG |
| Tas2r135 | Taste receptor, type 2, member 135 | NM_199159.1 | qPrimerDepot | F: TCAGGTACTGGATGTGGCAG |
|  |  |  |  | R: CAGCAGCCCCTCTTTATCAC |
| Tas2r143 | Taste receptor, type 2, member 143 | NM_001001452.1 | qPrimerDepot | F: CTTATTGGCATCCTCTGGGA |
|  |  |  |  | R: AGGGTGGGAGAAAGAGGAGA |
