# Supplementary material for: Expression, Regulation and Putative Nutrient-Sensing Function of Taste GPCRs in the Heart
Source: PLoS One. 2013 May 15;8(5):e64579. doi: 10.1371/journal.pone.0064579 (PMC3655793; doi:10.1371/journal.pone.0064579)
Supplement: Table S2 — In situ hybridization probe primer sequences, including T7 and T3 RNA polymerase binding sites. (DOCX) [file pone.0064579.s007.docx]

**Table S2: *In situ* hybridization probe primer sequences, including T7 and T3 RNA polymerase binding sites**

| Gene | 5' 🡪 3' forward with T7 | 5' 🡪 3' reverse with T3 | Product length |
| --- | --- | --- | --- |
| Tas1r1 | TAATACGACTCACTATAGGGGCCAAGCGCAAGTTCCCGT | AATTAACCCTCACTAAAGGGGGCTCCAAGCGTGGGCATGT | 610 |
| Tas1r3 | TAATACGACTCACTATAGGGCCCCGTGTGCTGGCTGTCAT | AATTAACCCTCACTAAAGGGGCAGCTAGGGCAAGGCGAGT | 632 |
| Tas2r120 | TAATACGACTCACTATAGGGTGCTCTTGCCATCTCCAGAATTGGT | AATTAACCCTCACTAAAGGGGGAAACCACAGTTTGCAAGGCCC | 553 |
| Tas2r121 | TAATACGACTCACTATAGGGTGCTGACAAACTGTATTGCTTGGCT | AATTAACCCTCACTAAAGGGAGGGTCTCGTTCCCCTCTGGA | 584 |
