# Supplementary material for: Expression, Regulation and Putative Nutrient-Sensing Function of Taste GPCRs in the Heart
Source: PLoS One. 2013 May 15;8(5):e64579. doi: 10.1371/journal.pone.0064579 (PMC3655793; doi:10.1371/journal.pone.0064579)
Supplement: Table S3 — RT-qPCR experiments were performed in the presence and absence of reverse transcriptase following DNase treatment to confirm the specific amplification of cDNA, in contrast to genomic DNA. (DOCX) [file pone.0064579.s008.docx]

**Table S3: RT-qPCR experiments were performed in the presence and absence of reverse transcriptase following DNase treatment to confirm the specific amplification of cDNA, in contrast to genomic DNA.**

|  | With reverse transcriptase | | | Without reverse transcriptase | | |
| --- | --- | --- | --- | --- | --- | --- |
| Gene symbol | Mean Ct | SEM | n^a^ | Mean Ct | SEM | n^b^ |
| Agtr1a | 28.71 | 0.36 | 4 | 37.22 | 0.92 | 4 (9/12) |
| Tas1r1 | 30.06 | 0.22 | 4 | N.D.^c^ |  | 4 (0/12) |
| Tas1r3 | 31.13 | 0.15 | 4 | 38.52 | 0.14 | 4 (2/12) |
| Tas2r108 | 32.30 | 0.22 | 4 | 35.26 | 0.7 | 4 (3/12) |
| Tas2r120 | 29.60 | 0.46 | 4 | 34.23 |  | 4 (3/12) |
| Tas2r121 | 29.57 | 0.52 | 4 | 34.83 | 1.09 | 4 (5/12) |
| Tas2r126 | 27.77 | 0.27 | 4 | 35.70 | 0.42 | 4 (4/12) |
| Tas2r135 | 28.91 | 0.22 | 4 | 36.77 |  | 4 (3/12) |
| Tas2r137 | 31.72 | 0.23 | 4 | 36.71 | 1.8 | 4 (5/12) |
| Tas2r143^d^ | 27.39 | 0.24 | 4 | 34.80 | 0.79 | 4 (3/12) |

^a^ Four samples were assayed in triplicate in the presence of reverse transcriptase. All 12 replicates amplified.

^b^ Four samples were assayed in triplicate in the absence of reverse transcriptase. Numbers in brackets denote the number of replicates where any amplification occurred, albeit at significantly later Ct values than observed in +RT samples.

^c^ No amplification detected.

^d^ For a representative amplification plot, see figure S1.
